# Interval Type-II Fuzzy Fault-Tolerant Control for Constrained Uncertain 2-DOF Robotic Multi-Agent Systems with Active Fault Detection

**DOI:** 10.3390/s23104836

**Published:** 2023-05-17

**Authors:** Wen Yan, Haiyan Tu, Peng Qin, Tao Zhao

**Affiliations:** College of Electrical Engineering, Sichuan University, Chengdu 610065, China; yanwenessay@stu.scu.edu.cn (W.Y.); qinpeng1@stu.scu.edu.cn (P.Q.); zhaotaozhaogang@scu.edu.cn (T.Z.)

**Keywords:** active fault detection, adaptive fuzzy fault-tolerant control, multi-agent systems

## Abstract

This study proposed a novel adaptive interval Type-II fuzzy fault-tolerant control for constrained uncertain 2-DOF robotic multi-agent systems with an active fault-detection algorithm. This control method can realize the predefined-accuracy stability of multi-agent systems under input saturation constraint, complex actuator failure and high-order uncertainties. Firstly, a novel active fault-detection algorithm based on pulse-wave function was proposed to detect the failure time of multi-agent systems. To the best of our knowledge, this was the first time that an active fault-detection strategy had been used in multi-agent systems. Then, a switching strategy based on active fault detection was presented to design the active fault-tolerant control algorithm of the multi-agent system. In the end, based on the interval type-II fuzzy approximated system, a novel adaptive fuzzy fault-tolerant controller was proposed for multi-agent systems to deal with system uncertainties and redundant control inputs. Compared with other relevant fault-detection and fault-tolerant control methods, the proposed method can achieve predefinition of stable accuracy with smoother control input. The theoretical result was verified by simulation.

## 1. Introduction

In recent years, multi-agent systems have been widely used in robots, factories, laboratories and networks [1,2,3,4]. However, because of actuator failure and system uncertainty, intelligent fault-tolerant control of multiple agents has become a research hotspot [5,6].

The existing fault-tolerant control strategies are mainly divided into passive fault-tolerant control strategies and active ones [7,8,9]. Most passive fault-tolerant control methods are based on robust control strategy, but this control strategy is often conservative and requires prior fault information [7]. In order to solve the defect that the fault information in passive fault-tolerant control needs prior information, the active fault-tolerant control was proposed by adding a fault-detection and diagnosis module [10]. This strategy can realize the online reconstruction of the controller without prior fault information. An active fault-tolerant control method was proposed by integrating detection, diagnosis and controller reconstruction, but it may be unstable in the detection and diagnosis stages [11]. In order to solve this stability problem, an active fault-tolerant control method was proposed as a robust control idea to deal with the conflict between stabilization and restructuring [12]. After that, active fault-tolerant control has been widely used in various mechanical control systems. Active fault-tolerant control was applied to unmanned aerial vehicles (UAVs), which achieved the rapid stability of the control system under actuator failure [13]. In order to solve the stability problem of underwater robots under actuator fault conditions, an active fault-tolerant control was used to realize the stability of the closed-loop system [14,15]. In order to improve the fault-tolerant control performance of the manipulator, an active fault-tolerant control based on redundant motors was proposed to reduce the structural complexity [16]. In order to improve the performance of fault-tolerant controls under noisy conditions, fault detection was often used [17,18,19,20,21]. With the development of fault-detection technology, it was applied in many fields [22,23,24,25,26]. However, traditional active fault-tolerant controls mainly relied on a passive detection mechanism. In systems with complex uncertainties and faults, the passive detection mechanism may not be sensitive enough to detect faults. In order to improve the sensitivity of the detection stage, an active fault-detection algorithm was proposed by adding auxiliary input signals [27]. Since the active fault-detection algorithm can identify more complex and variable faults, it has been studied by some scholars [28,29,30]. However, the existing active detection algorithms are mainly for single-agent systems and few are for multi-agent systems. The subsystem faults of multi-agent systems are often different and the sensors are interfered with by multiple subsystems. As a result, the detection mechanism may be insensitive to faults and thus trigger incorrectly. Furthermore, the constraints of multi-agent controllers are also very complex [31] and the control input saturation is not considered in most fault-tolerant control methods for multi-agent systems. Hence, it is a challenge to detect complex faults in multi-agent systems.

The actual control system often contains complex and changeable uncertainties, and fuzzy logic systems are often used to approximate system uncertainty because of their good approximation performance [32,33,34,35]. It is often difficult to deal with high-order uncertainties with traditional fuzzy logic systems, so type-II fuzzy control was proposed to improve the approximation performance to complex uncertainties [36]. The calculation of traditional Type-II fuzzy logic systems is often slow, so an interval Type-II fuzzy logic system was applied to the design of the controller [37]. In order to make the fuzzy logic system approximate the rapidly changing uncertainty, an adaptive fuzzy control strategy was proposed based on the adaptive adjustment of weight parameters [38]. Based on that, the weight adaptive law was applied to interval Type-II fuzzy control to deal with high-order uncertainties and changeable uncertainties [39]. By considering the fault-tolerant control algorithm, an interval Type-II fuzzy fault-tolerant control was proposed to deal with system faults and system uncertainties at the same time [40]. However, most existing interval Type-II fuzzy fault-tolerant control methods are based on passive fault detection [40,41,42]. This passive detection has difficulty distinguishing between faults and the uncertainty of multi-agent systems, which is caused by the strong coupling of multiple subsystems. This problem may make it difficult for the fuzzy system to approximate the actual uncertainty and affect the system control performance. Meanwhile, the active fault-tolerant control strategy of the multi-agent system can also lead to fast changes in uncertainty. Therefore, it is valuable to study adaptive interval Type-II fuzzy fault-tolerant controls for multi-agent systems.

Motivated by the above-mentioned problem, we propose a novel adaptive interval Type-II fuzzy fault-tolerant control for the proposed multi-agent systems based on active fault detection. This control method can realize the predefined-accuracy stability of multi-agent systems under input saturation, complex actuator failure and high-order uncertainties. The main innovative contributions are listed in the following:

(1) To the best of our knowledge, active fault detection of multi-agent systems is realized for the first time. Compared with the existing passive fault-detection methods, the novel active detection algorithm can resist more topology communication interference than passive detection.

(2) An improved fault-tolerant control algorithm of multi-agent systems was designed by the novel active fault-detection switching strategy. Compared with the existing passive fault-tolerant control methods, the proposed method can handle more serious and complex actuator failures in multi-agent systems.

(3) Based on the interval Type-II fuzzy approximated system, a novel adaptive fuzzy fault-tolerant controller was proposed for multi-agent systems to deal with high-order uncertainties and redundant control inputs. Compared with other fault-tolerant control methods, the proposed method can achieve predefinition of stable accuracy.

In the following, Section 2 presents the preliminaries. Section 3 is the problem description. Section 4 presents the results. Section 5 is the simulation analysis. Section 6 is the conclusion.

## 2. Preliminaries

**Assumption** **1** ([43,44,45])**.** *Assume that only the failures given in Definition 2 occur during system operation and no additional failures occur. The soundness of the system can be ensured in this study.*

**Lemma** **1** ([32])**.** *Consider a continuous function: $f\left(x\right):D_{f}\to R$ and $D_{f}$ is the compact set. Then, f(x) can be approximated by an interval Type-II fuzzy logic system $w^{T}\beta \left(x\right)$ with arbitrary small error δ:*

(1) $$\begin{array}{c} \left|f\left(x\right)-w^{T}\beta \left(x\right)\right|\leq \delta \end{array}$$

**Figure 1:**
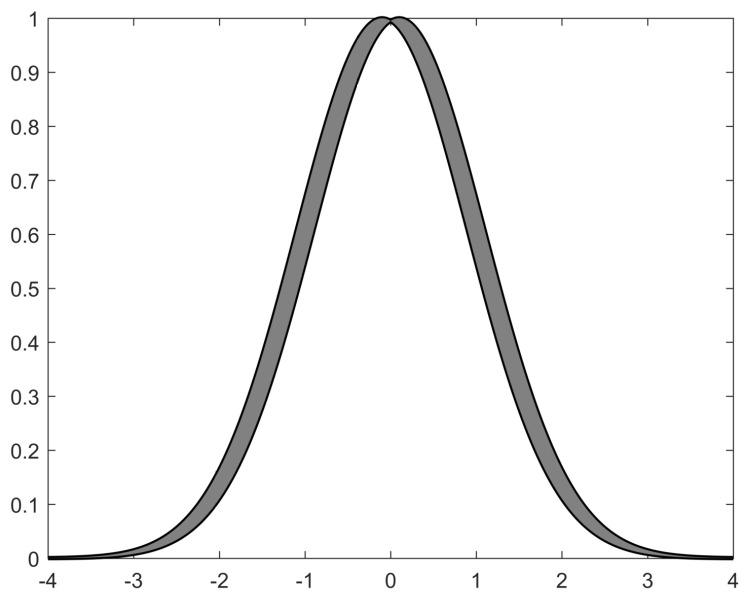
Interval Type-II fuzzy membership function.

*where $\hat{w}\in R^{r}$ is the adaptive weight parameter vector. $\hat{w}\in R^{r}$ is the expected weight parameter vector. $\beta \left(x\right)\in R^{\prod_{i=1}^{n}r_{i}}$ is a basis function as shown in Figure 1, which can be expressed as:*

(2) $$\beta_{r}\left(x\right)=\beta_{r}^{L}\left(x\right){\underline{\vartheta }}_{r}+\beta_{r}^{U}\left(x\right){\bar{\vartheta }}_{r}$$

*in which*

(3) $$\begin{array}{c} \beta_{r}^{LU}\left(x\right)=\left[\prod_{\phi }^{h=1}\mu_{{\tilde{A}}_{h}^{r}}^{L}\left(x\right),\prod_{\phi }^{h=1}\mu_{{\tilde{A}}_{h}^{r}}^{U}\left(x\right)\right]\\ \;\;\;\;\;\;\;\;\;\;\;\;\;=\left[\beta_{r}^{L}\left(x\right),\beta_{r}^{U}\left(x\right)\right] \end{array}$$

*here, ${\underline{\vartheta }}_{r}+{\bar{\vartheta }}_{r}=1$. $\mu_{{\tilde{A}}_{h}^{r}}^{L}\left(x\right)$ and $\mu_{{\tilde{A}}_{h}^{r}}^{U}\left(x\right)$ are the lower and upper membership grades:*

(4) $$\left\{\begin{array}{c} \mu_{{\tilde{A}}_{h}^{r}}^{L}\left(x_{h}\right)=exp\left(-\frac{1}{2}\left(\frac{x_{h}-m_{h,r}^{L}}{\sigma_{h}^{r}}\right){}^{2}\right)\\ \mu_{{\tilde{A}}_{h}^{r}}^{U}\left(x_{h}\right)=exp\left(-\frac{1}{2}\left(\frac{x_{h}-m_{h,r}^{U}}{\sigma_{h}^{r}}\right){}^{2}\right) \end{array}\right.$$

*with the following fuzzy rules:*

(5) $$\begin{array}{c} Rule^{r}:\;IF\;x_{1}\;is\;{\tilde{A}}_{1}^{r}\;and\;\cdots \;and\;x_{n}\;is\;{\tilde{A}}_{n}^{r},\\ \;\;\;\;\;\;\;\;\;\;\;\;\;\;Then\;w^{T}\beta \left(x\right)\;is\;{\tilde{B}}^{r} \end{array}$$

*Here, the fuzzy set is considered as the complete and continuous set [46,47,48,49,50,51].*

## 3. Problem Description

Multi-agent systems are based on graph theory and the relevant background is described in Appendix A.

**Definition** **1.** *A 2-DOF robotic multi-agent system is defined with a leader and N (N≥2) followers:*

(6) $$\left\{\begin{array}{c} {\dot{q}}_{i,1}=q_{i,2}\\ {\dot{q}}_{i,2}=f_{i}\left(q_{i,1},q_{i,2}\right)+\Delta f_{i}\left(q_{i,1},q_{i,2}\right)+\left(g_{i}\left(q_{i,1}\right)+\Delta g_{i}\left(q_{i,1}\right)\right)\left(u_{i}+u_{a_{i}}\right) \end{array}\right.$$

*and the dynamic model of the leader is described as follows (i=l):*

(7) $$\left\{\begin{array}{c} {\dot{q}}_{l,1}=q_{l,2}\\ {\dot{q}}_{l,2}=f_{l}\left(q_{l,1},q_{l,2}\right)+\Delta f_{l}\left(q_{l,1},q_{l,2}\right)+\left(g_{i}\left(q_{i,1}\right)+\Delta g_{i}\left(q_{i,1}\right)\right)\left(u_{l}+u_{a_{l}}\right) \end{array}\right.$$

*where $q_{i,1}={\left[q_{i,1,1},\ldots ,q_{i,1,n}\right]}^{T}$ and $q_{i,2}={\left[q_{i,2,1},\ldots ,q_{i,2,n}\right]}^{T}$. $u_{i}={\left[u_{i,1},\ldots ,u_{i,n}\right]}^{T}$ represents the main control input and $u_{a_{i}}$ represents the redundant control input. $f_{i}\left(q_{i,1},q_{i,2}\right)=-M_{i}{\left(q_{i,1}\right)}^{-1}\left(C_{i}\left(q_{i,1},q_{i,2}\right)q_{i,2}+G_{i}\left(q_{i,1}\right)\right)$, in which $M_{i}\left(q_{i,1}\right)$ is the symmetric inertia matrix, $C_{i}\left(q_{i,1},q_{i,2}\right)$ is the centripetal and Coriolis torques matrix and $G_{i}\left(q_{i,1}\right))$ is the gravitational torque. $g_{i}\left(q_{i,1}\right)=M_{i}{\left(q_{i,1}\right)}^{-1}$. $\Delta f_{i}\left(q_{i,1},q_{i,2}\right)$ and $\Delta g_{i}\left(q_{i,1}\right)$ denote the unknown uncertainties caused by parameter perturbation and modeling uncertainty. When the subscript i is replaced by l, the symbolic meaning is that of the leader. Uncertainty from multiple sources can be called high-order uncertainty.*

The tracking error of *i*th follower in (6) is defined as:

(8) $$\begin{array}{c} z_{i,1}=\sum_{h=1}^{N}a_{ih}\left(q_{i,1}-q_{h,1}\right)+b_{i}\left(q_{i,1}-q_{l,1}\right) \end{array}$$

where $a_{ih}$ and $b_{i}$ are the weight parameters.

**Condition** **1.** The input constraint is $\left|u_{i,k}\right|\leq U$, in which *U* is the known and bounded constant.

**Lemma** **2.** *In order to achieve Condition 1, the actual control input $u_{i,k}$ can be designed by [52] as:*

(9) $$u_{i,k}=U\tanh\left(\frac{v_{i,k}}{U}\right)$$

*where $v_{i,k}=u_{i,k}\left(v_{i,k}\right)+e\left(v_{i,k}\right)$. $e_{i,k}\left(v_{i,k}\right)\leq E$ and E is a bounded unknown constant.*

**Definition** **2.** *The faults of sub-systems for multi-agent systems are often various, so their overall fault situation is complex. The actuator fault of subsystem [5] is considered as the following:*

(10) $$\begin{array}{c} u_{f_{i,k}}=\Psi_{i,k}\left(q_{i,1,k},t\right)u_{i,k}+\Phi_{i,k}\left(t\right) \end{array}$$

*in which*

(11) $$\Psi_{i,k}\left(q_{i,1,k},t\right)=\left\{\begin{array}{cc} exp\left(-\eta_{i,k}t+\varpi_{i,k}\right)+0.1sin\left(q_{i,1,k}\right) & ,t\geq t_{a_{i,k}}\\ 1 & ,t<t_{a_{i,k}} \end{array}\right.$$

*where $\eta_{i,k}t_{act}=\varpi_{i,k}$. $\eta_{i,k}$ and $\varpi_{i,k}$ are the positive parameters. $t_{a_{i,k}}$ is the failure time of actuator. $\Phi_{i,k}\left(t\right)$ is considered to be zero in this paper.*

The control objective is to make the tracking error of the system converge to the predefined accuracy † before and after the fault occurs.

## 4. Results

**Figure 2:**
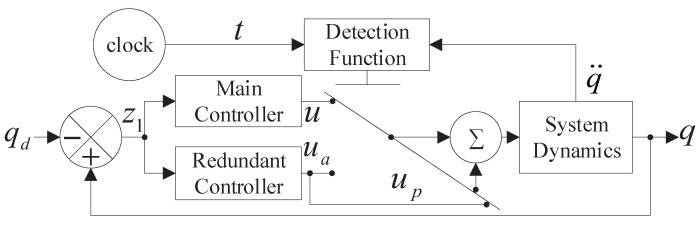
The controller structure block diagram of the agent.

The design scheme of the proposed controller is shown in Figure 2. Assumption 1 is satisfied and the system is considered optimized [53,54,55,56,57].

### 4.1. Active Fault Detection and Fault-Tolerant Control

The auxiliary input signal of redundant controller $u_{p}$ is considered as a pulse-wave function:

(12) $$u_{p}\left(t\right)=\left\{\begin{array}{cc} U_{m} & ,\;\kappa t_{p}+t_{0}\leq t\leq \kappa t_{p}+t_{0}+\Delta t\\ 0 & ,\;others \end{array}\right.$$

where κ=1,2,3…. $U_{m}={\left[U_{m},\ldots ,U_{m}\right]}^{T}$ is the pulse amplitude vector. $t_{p}$ is the pulse repetition period. $t_{0}$ is the start time of detection. Δt is the pulse width.

By observing (6) and (7), it is clear that observable information ${\dot{q}}_{i,2}$ is more sensitive to the change of control input $u_{a_{i}}$ than other observable information $q_{i,2}$ and $q_{i,1}$. Hence, a novel active detection algorithm is designed as follows.

When flag=1 in Algorithm 1, the system is judged to be faulty. Then, the following improved active fault-tolerant control algorithm is activated:
**Algorithm 1** Active fault-detection algorithm (i=l,1,2,3,…)Initial stage       $u_{i}=U\tanh\left(\frac{v_{i}}{U}\right)$       $u_{a_{i}}=u_{p_{i}}$Initial Y (A fault threshold parameter)Global Flag The 1th detection cycle(2)    $If\;t\approx t_{p}+t_{0}+\Delta t$       $If\;∥{\dot{q}}_{i,2}\left(t\right)-{\dot{q}}_{i,2}\left(t-\Delta t\right)∥>∥{\dot{q}}_{i,2}\left(t-t_{1}\right)-{\dot{q}}_{i,2}\left(t-t_{1}-\Delta t\right)∥+Y$           Flag=1       end    end⋮The κ−1th detection cycle(κ)    $If\;t\approx \kappa t_{p}+t_{0}+\Delta t$       $If\;∥{\dot{q}}_{i,2}\left(t\right)-{\dot{q}}_{i,2}\left(t-\Delta t\right)∥>∥{\dot{q}}_{i,2}\left(t-t_{p}\right)-{\dot{q}}_{i,2}\left(t-t_{p}-\Delta t\right)∥+Y$           Flag=1       end    end

### 4.2. Main Controller Design

#### 4.2.1. Main Controller Design of Leader

The virtual error $z_{l,2}$ can be designed as:

(13) $$\left\{\begin{array}{c} z_{l,1}=q_{l,1}-q_{d_{l,1}}\\ z_{l,2}=q_{l,2}-\alpha_{l,1} \end{array}\right.$$

where $\alpha_{l,1}$ is virtual control.

The uncertainty of the system can be approximated by a fuzzy logic system [58,59] and then an approximation-based controller can be designed as follows.

**Theorem** **1.** *The leader system in Definition 1—(7) can be controlled by the following controller with a predefined accuracy $B_{l}=\left\{\left.z_{l,1}\right|∥z_{l,1}∥\leq †\right\}$:*

(14) $$\left\{\begin{array}{ccc} \alpha_{l,1} & = & -\frac{\left(2{†}^{2}+{\hat{k}}_{l}\right)z_{l,1}}{2{†}^{2}}+{\dot{q}}_{d_{l,1}}\\ v_{l} & = & -g_{l}{\left(q_{l,1}\right)}^{-1}\left(f_{l}\left(q_{l,1},q_{l,2}\right)+\frac{1}{2}{\hat{\theta }}_{l}^{T}\psi_{l}\left(q_{l,1},q_{l,2}\right)z_{l,2}+z_{l,1}+\frac{3}{2}z_{l,2}-{\dot{\alpha }}_{l,1}\right)\\ u_{l} & = & U\tanh\left(\frac{v_{l}}{U}\right) \end{array}\right.$$

*and the adaptive law is*

(15) $$\begin{array}{c} {\dot{\hat{\theta }}}_{l}=\frac{z_{l,2}^{T}z_{l,2}\psi_{l}\left(q_{l,1},q_{l,2}\right)}{2} \end{array}$$

*and the adaptive law is*

(16) $$\begin{array}{c} {\dot{\hat{k}}}_{l}=\frac{z_{l,1}^{T}z_{l,1}}{2\xi_{l}^{2}{†}^{2}} \end{array}$$

*where ${\hat{\theta }}_{l}={\left[{\hat{\theta }}_{l,1},\ldots ,{\hat{\theta }}_{l,n}\right]}^{T}$ is the adaptive parameter vector. ${\hat{k}}_{l}$ is an adaptive parameter and $\xi_{l}$ is a positive parameter. $\psi_{l,k}\left(q_{l,1},q_{l,2}\right)=\beta_{l,k}{\left(q_{l,1},q_{l,2}\right)}^{T}\beta_{l,k}\left(q_{l,1},q_{l,2}\right)$, and $\psi_{l}\left(q_{l,1},q_{l,2}\right)={\left[\psi_{l,1}\left(q_{l,1},q_{l,2}\right),\ldots ,\psi_{l,n}\left(q_{l,1},q_{l,2}\right)\right]}^{T}$.* † *is the accuracy parameter.*

The proof of Theorem 1 is given in Appendix B.

#### 4.2.2. Main Controller Design of Follower

The virtual error $z_{i,2}$ can be designed as:

(17) $$z_{i,2}=q_{i,2}-\alpha_{i,1}$$

where $\alpha_{i,1}$ is the virtual control.

**Theorem** **2.** *The follower systems in Definition 1—(6) can be controlled by the following controller with a predefined accuracy $B_{i}=\left\{\left.z_{i,1}\right|∥z_{i,1}∥\leq †\right\}$:*

(18) $$\left\{\begin{array}{ccc} \alpha_{i,1} & = & \frac{1}{\left(b_{i}+\sum_{h=1}^{N}a_{ih}\right)}\left(b_{i}q_{l,2}+\sum_{h=1}^{N}a_{ih}q_{h,2}-\frac{\left(2{†}^{2}+{\hat{k}}_{i}\right)z_{i,1}}{2{†}^{2}}\right)\\ v_{i} & = & -g_{i}{\left(q_{i,1}\right)}^{-1}\left(f_{i}\left(q_{i,1},q_{i,2}\right)+\frac{1}{2}{\hat{\theta }}_{i}^{T}\psi_{i}\left(q_{i,1},q_{i,2}\right)z_{i,2}\right.\\ & & +\left.\left(b_{i}+\sum_{h=1}^{N}a_{ih}\right)z_{i,1}+\frac{3}{2}z_{i,2}-{\dot{\alpha }}_{i,1}\right)\\ u_{i} & = & U\tanh\left(\frac{v_{i}}{U}\right) \end{array}\right.$$

*and the adaptive law is*

(19) $$\begin{array}{c} {\dot{\hat{\theta }}}_{i}=\frac{z_{i,2}^{T}z_{i,2}\psi_{i}\left(q_{i,1},q_{i,2}\right)}{2} \end{array}$$

*and the adaptive law is*

(20) $$\begin{array}{c} {\dot{\hat{k}}}_{i}=\frac{z_{i,1}^{T}z_{i,1}}{2\xi_{i}^{2}{†}^{2}} \end{array}$$

*where ${\hat{\theta }}_{i}={\left[{\hat{\theta }}_{i,1},\ldots ,{\hat{\theta }}_{i,n}\right]}^{T}$ is the adaptive parameter vector. ${\hat{k}}_{i}$ is an adaptive parameter and $\xi_{i}$ is a positive parameter. $\psi_{i,k}\left(q_{i,1},q_{i,2}\right)=\beta_{i,k}{\left(q_{i,1},q_{i,2}\right)}^{T}\beta_{i,k}\left(q_{i,1},q_{i,2}\right)$ and $\psi_{i}\left(q_{i,1},q_{i,2}\right)={\left[\psi_{i,1}\left(q_{i,1},q_{i,2}\right),\ldots ,\psi_{i,n}\left(q_{i,1},q_{i,2}\right)\right]}^{T}$.* † *is the accuracy parameter.*

The proof of Theorem 2 is given in Appendix C.

### 4.3. Redundant Controller Design

**Theorem** **3.** *According to Algorithm 2, the redundant controller can be designed as follows (i=l,1,2,3,…):*

(21) $$\left\{\begin{array}{c} u_{a_{i}}=u_{p_{i}}\;\;\;\;\;\;\;\;\;\;\;\;\;\;,\;flag=0\\ u_{a_{i}}=U\tanh\left(\frac{v_{l}}{U}\right)\;,\;flag=1 \end{array}\right.$$

*where $u_{p_{i}}$ is from (12). When flag=1, the proof of Theorem 3 is similar to Theorems 1 and 2. The mathematical principle is that the value of $u_{i}$ and $u_{a_{i}}$ can be exchanged to obtain a symmetric stability result. According to (A10), (A21) and (21), it is clear that $z_{i,1}$ can converge to a neighborhood of zero $B_{i}=\left\{\left.z_{i,1}\right|∥z_{i,1}∥\leq †\right\}$ after controller reconstruction.*

**Algorithm 2** Active fault-tolerant control algorithm (i=l,1,2,3,…)
Initial stage       $u_{i}=U\tanh\left(\frac{v_{i}}{U}\right)$       $u_{a_{i}}=u_{p_{i}}$Switching stage [60]    Ifflag=1       $u_{i}=0$       $u_{a_{i}}=U\tanh\left(\frac{v_{i}}{U}\right)$    end


### 4.4. Stability Analysis of the System

The Lyapunov functions in (A1) $V_{l,1}$, (A3) $V_{l,2}$, (A11) $V_{i,1}$ and (A14) $V_{i,2}$ are considered to verify the stability of system.

No-fault stage: According to Theorems 1 and 2, it can be deduced that ${\dot{V}}_{l,1}\leq 0$, ${\dot{V}}_{l,2}\leq 0$, ${\dot{V}}_{i,1}\leq 0$ and ${\dot{V}}_{i,2}\leq 0$ at $q_{i,1}\in B_{i}$. Hence, the system is predefined-accuracy stable.

Fault and no-switching stage: According to Algorithm 1, if flag=0, ${\dot{q}}_{i,2}$ is small and bounded. Then, $q_{i,2}$ and $q_{i,1}$ are bounded if the operation time is finite. Next, by considering (8), (13), (14) and (18), and $q_{d_{i,1}}$ is bounded, it can be deduced that each virtual error z is bounded. Then, the Lyapunov functions $V_{l,1}=z_{l,1}^{T}z_{l,1}$, $V_{l,2}=z_{l,2}^{T}z_{l,2}$, $V_{i,1}=z_{i,1}^{T}z_{i,1}$ and $V_{i,2}=z_{i,2}^{T}z_{i,2}$ are also bounded. Hence, according to the above-mentioned bounded inference and Barbalat stability theorem, the system is Lyapunov stable. The steady-state accuracy at this stage can be adjusted by a fault threshold parameter *Y* in Algorithm 1.

Switching stage: If the switching is considered as a momentary event [61] and Condition 1 is considered, (A4) can be rewritten as:

(22) $$\begin{array}{c} {\dot{V}}_{l,2}\leq -\frac{\left(2{†}^{2}+{\hat{k}}_{l}\right)z_{l,1}^{T}z_{l,1}}{2{†}^{2}}+z_{l,1}^{T}z_{l,2}+z_{l,2}^{T}\left[f_{l}\left(q_{l,1},q_{l,2}\right)+\Delta f_{l}\left(q_{l,1},q_{l,2}-{\dot{\alpha }}_{l,1}\right)\right]\\ \;\;\;\;\;\;\;\;\;\;\;-{\tilde{\theta }}_{l}^{T}{\dot{\hat{\theta }}}_{l}-\xi_{l}^{2}{\tilde{k}}_{l}{\dot{\hat{k}}}_{l}+\left|z_{l,2}^{T}\right|\left|g_{l}\left(q_{l,1}\right)+\Delta g_{l}\left(q_{l,1}\right)\right|\left(\left|u_{i}\right|+\left|u_{a_{l}}\right|\right)\\ \;\;\;\;\;\;\;\leq -\frac{\left(2{†}^{2}+{\hat{k}}_{l}\right)z_{l,1}^{T}z_{l,1}}{2{†}^{2}}+z_{l,1}^{T}z_{l,2}+z_{l,2}^{T}\left[f_{l}\left(q_{l,1},q_{l,2}\right)+\Delta f_{l}\left(q_{l,1},q_{l,2}-{\dot{\alpha }}_{l,1}\right)\right]\\ \;\;\;\;\;\;\;\;\;\;\;-{\tilde{\theta }}_{l}^{T}{\dot{\hat{\theta }}}_{l}-\xi_{l}^{2}{\tilde{k}}_{l}{\dot{\hat{k}}}_{l}+\left|z_{l,2}^{T}\right|\left|g_{l}\left(q_{l,1}\right)+\Delta g_{l}\left(q_{l,1}\right)\right|\left(2U\right) \end{array}$$

Since the virtual errors of the system z in the fault and no-switching stage are bounded, $\dot{V}$ is bounded. Assuming that the switching time is a very small constant τ. By considering that the Lyapunov functions *V* in the fault and no-switching stage are also bounded, then $V\left(t-\tau \right)+\dot{V}\tau =V\left(t\right)$ is bounded. Hence, the leader system is also Lyapunov stable by considering (22).

Redundant control stage: According to Theorem 3, the system is predefined-accuracy stable at flag=1.

**Remark** **1.** *Based on the proposed active fault-detection and redundant fault-tolerance mechanism, the Lyapunov stability analysis of this system can be regarded as continuous. Hence, compared with other existing fault-tolerant control methods, the proposed method can achieve predefinition of stable accuracy.*

## 5. Simulation

**Figure 3:**
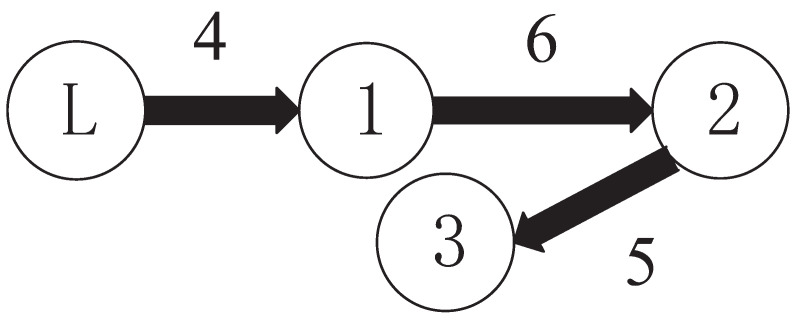
The communication topology of MASs.

The following simulations are carried out on MATLAB R2016a with a variable simulation step of ode45, $10^{-5}$ relative tolerance and auto other additional options. A small image embedded in a large image is a local magnification of a large image with the same time scale. Section 5.1 presents the validation simulations of the proposed controller for the single-agent system. Section 5.2 presents the validation simulations of the proposed controller for multi-agent systems with different actuator subsystem faults. Section 5.3 presents comparative simulations between the proposed method and recent passive fault-tolerant methods. Section 5.4 presents the comparative simulations between the proposed method and recent active fault-tolerant methods. This active fault-tolerant method adopts passive fault detection. The structure of multi-agent systems is shown in Figure 3; the subsystem is modeled as the following 2-DOF robot arm system. The advantages of the proposed method can be verified by comparative simulation.

According to recent robot studies [62,63,64], the dynamics of simplified 2-DOF robot arm system are modeled as follows:

(23) $$M\left(q\right)\ddot{q}+C\left(q,\dot{q}\right)\dot{q}+G\left(q\right)+D\left(q\right)=\tau$$

in which,
 

$$\begin{array}{l} M\left(q\right)=\left[\begin{array}{cc} \left(m_{1}+m_{2}\right)l_{1}^{2}+m_{2}l_{2}^{2}+2m_{2}l_{1}l_{2}\cos\left(q_{2}\right) & m_{2}l_{2}^{2}+m_{2}l_{1}l_{2}\cos\left(q_{2}\right)\\ m_{2}l_{2}^{2}+m_{2}l_{1}l_{2}\cos\left(q_{2}\right) & m_{2}l_{2}^{2} \end{array}\right],\\ C\left(q,\dot{q}\right)=\left[\begin{array}{cc} -2m_{2}l_{1}l_{2}{\dot{q}}_{2}\sin\left(q_{2}\right) & -m_{2}l_{1}l_{2}\left({\dot{q}}_{1}+{\dot{q}}_{2}\right)\sin\left(q_{2}\right)\\ m_{2}l_{1}l_{2}{\dot{q}}_{1}\sin\left(q_{2}\right) & 0 \end{array}\right],\\ G\left(q\right)=\left[\begin{array}{c} \left(m_{1}+m_{2}\right)gl_{1}\cos\left(q_{1}\right)+m_{2}gl_{2}\cos\left(q_{1}+q_{2}\right)\\ m_{2}gl_{2}\cos\left(q_{1}+q_{2}\right) \end{array}\right],\\ D\left(q\right)=\left[\begin{array}{c} 0.1\sin\left(q_{1}\right)+0.1\cos\left(q_{2}\right)\\ 0.1\sin\left(q_{2}\right)+0.1\cos\left(q_{1}\right) \end{array}\right], \end{array}$$

and parameter perturbation is expressed as:

(24) $$\left\{\begin{array}{c} \Delta m_{1}=\pm 10\%\\ \Delta m_{2}=\pm 10\% \end{array}\right.$$

where D(q) is disturbance. g=9.8 kg/m${}^{2}$. Mass parameters are $m_{1}=1$ and $m_{2}=0.5$. Link lengths are $l_{1}=1$ and $l_{2}=0.5$.

The initial condition is $\left[\begin{array}{cccc} q_{1} & {\dot{q}}_{1} & q_{2} & {\dot{q}}_{2} \end{array}\right]=\left[\begin{array}{cccc} 0.5\pi & 0.5\pi & 0.5\pi & 0.5\pi \end{array}\right]$ and the joint angle command is $\left[\begin{array}{cc} q_{d_{1}} & q_{d_{2}} \end{array}\right]=\left[\begin{array}{cc} \sin(t)+0.5 & \sin(t)+0.5 \end{array}\right]$.

The singularity problem of Jacobian matrix M(q) is solved by the DLS method [65]:

(25) $${\left(M\left(q\right)\right)}^{-1}=\sum_{i=1}^{2}\frac{s_{i}}{{\left(s_{i}\right)}^{2}+{\left(\zeta_{i}\right)}^{2}}\psi_{i}\nu_{i}^{T}$$

in which

(26) $$\zeta_{i}=\left\{\begin{array}{c} 0.008\left(1+\cos\left(\frac{\pi s_{i}}{0.003}\right)\right)\;,\;\left|s_{i}\right|\leq 0.003\\ 0\;\;\;\;\;\;\;\;\;\;\;\;\;\;\;\;\;\;\;\;\;\;\;\;\;\;\;\;\;\;\;\;\;\;\;,\;others \end{array}\right.,$$

where [ν,s,ψ]=SVD(M(q)).

By considering one communication topology structure of MASs as shown in Figure 3, the weighted Laplacian matrix *L* and weighted adjacency matrix $W_{1}$ defined by [66] are shown in the following:

(27) $$\left[\begin{array}{ccc} 0 & 0 & 0\\ 6\times 1 & 0 & 0\\ 0 & 5\times 1 & 0 \end{array}\right],\left[\begin{array}{ccc} 0 & 0 & 0\\ 6\times -1 & 6\times 1 & 0\\ 0 & 5\times -1 & 5\times 1 \end{array}\right]$$

with a connected weight matrix of leader and followers

(28) $$\left[\begin{array}{ccc} 4\times 1 & 0 & 0\\ 0 & 0 & 0\\ 0 & 0 & 0 \end{array}\right]$$

The auxiliary input signal is selected as

(29) $$u_{p}\left(t\right)=\left\{\begin{array}{c} u_{m}\;,\;\kappa t_{p}+t_{0}\leq t\leq \kappa t_{p}+t_{0}+\Delta t\\ 0\;\;\;\;\;,\;others \end{array}\right.$$

where $U_{m}=1$, $t_{p}=2$, $t_{0}=0.5$, Δt=0.05.

The interval type 2 membership function with ${\underline{\vartheta }}_{r}={\bar{\vartheta }}_{r}=\frac{1}{2}$ is chosen as the Gaussian function:

(30) $$\left\{\begin{array}{c} \mu_{{\tilde{A}}_{h}^{r}}^{L}\left(x_{h}\right)=exp\left(-\frac{1}{2}\left(\frac{x_{h}-m_{h,r}^{L}}{\sigma_{h}^{r}}\right){}^{2}\right)\\ \mu_{{\tilde{A}}_{h}^{r}}^{U}\left(x_{h}\right)=exp\left(-\frac{1}{2}\left(\frac{x_{h}-m_{h,r}^{U}}{\sigma_{h}^{r}}\right){}^{2}\right) \end{array}\right.$$

where $\sigma_{h}^{r}=1$, $m_{h,r}^{L}=-2.1,-1.1,-0.1,0.9,1.9$, $m_{h,r}^{U}=-1.9,-0.9,0.1,1.1,2.1$.

### 5.1. Validation Simulations of the Proposed Controller for Single-Agent Systems

In this section, the validation simulation of the proposed method for uncertain single-agent systems is carried out with Condition 1. The model of the single-agent system is based on the leader model of topology-free communication. The initial detector parameter is Y=0.1. The initial parameters are †=0.1, U=30, ${\hat{\theta }}_{l}\left(0\right)=0$, ${\hat{k}}_{l}\left(0\right)=0.4$, $\xi_{l}=\sqrt{10}\times 10^{-3}$. The actuator fault parameters in Definition 2 are set as follows: $\eta_{l,1}=\eta_{l,2}=0.05$, $\varpi_{l,1}=\varpi_{l,2}=0.5$ and $t_{a_{l,1}}=t_{a_{l,2}}=10$.

**Figure 4:**
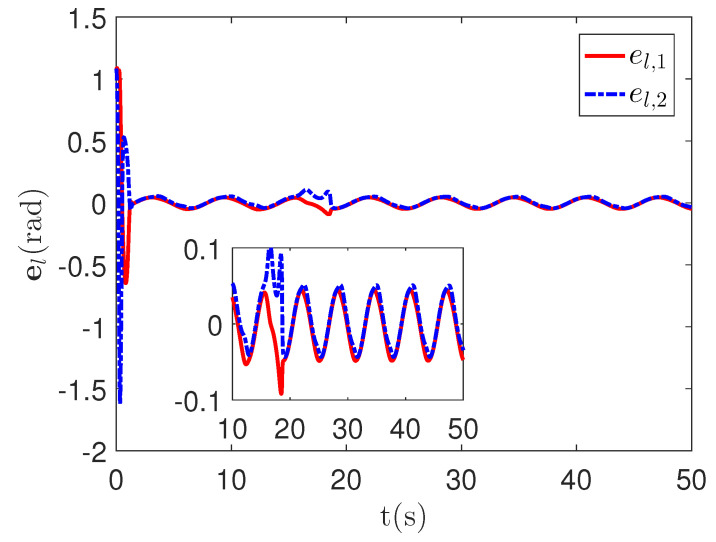
The tracking error curves $e_{l}=q_{l,1}-q_{d_{l,1}}$ of the proposed method.

**Figure 5:**
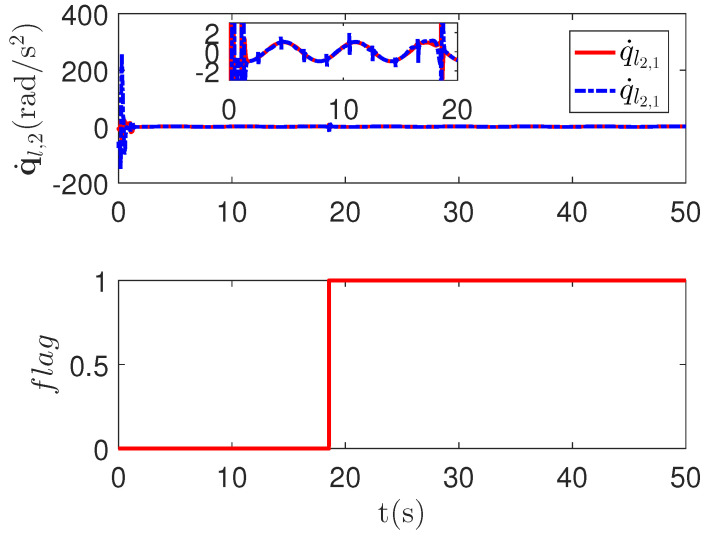
The joint angular acceleration curves ${\dot{q}}_{l,2}$ and fault-detection curve Flag of the proposed method.

**Figure 6:**
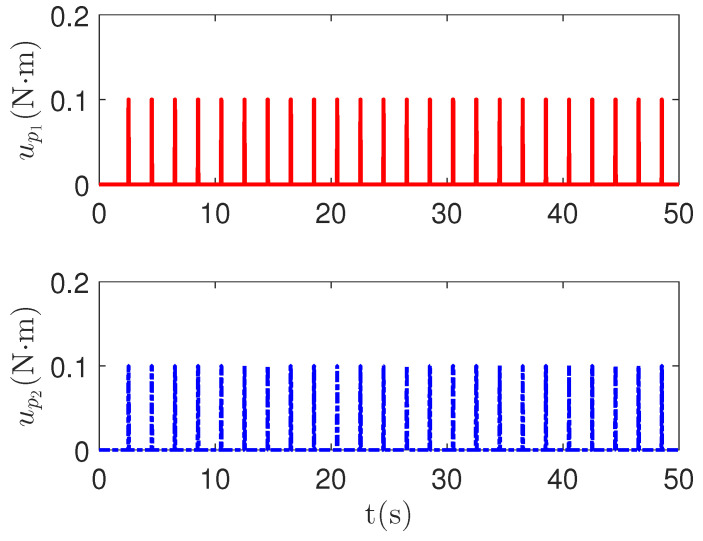
The pulse-wave function curves $u_{p}$ of the redundant control input $u_{a_{l}}$.

**Figure 7:**
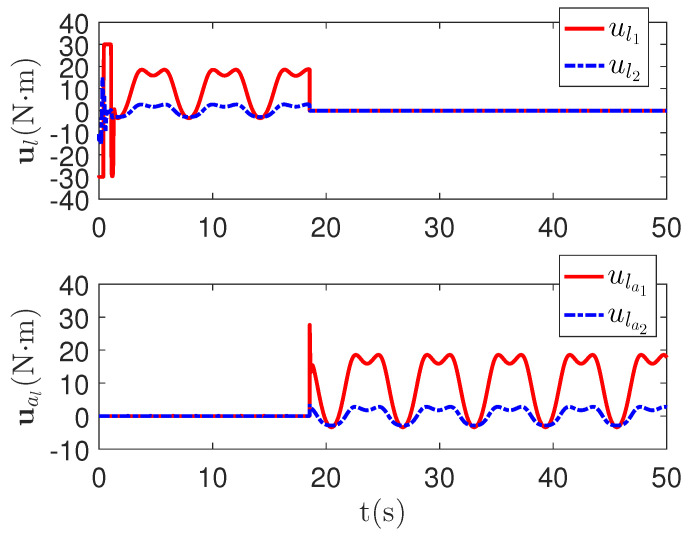
The main control input curves $u_{l}$ and redundant control input curves $u_{a_{l}}$ of the proposed method.

As shown in Figure 4, it is clear that the tracking error of the proposed method is about 0.05, which is less than the predefined accuracy †=0.1. By considering the simulation results in Figure 4 and Figure 5, there is no significant change in system tracking performance when the fault occurs at 10 s. The reason is the robustness of the control system. Therefore, the auxiliary input in Figure 6 is considered to be added to the control system. According to the results in Figure 5, when the sensor detects the occurrence of acceleration-level abnormal phenomena, Algorithm 1 judges the system failure flag=1 at about 18.55 s. Furthermore, as shown in Figure 7, the maximum absolute values of $u_{l}$ and $u_{a_{l}}$ are 30 and 27.63, respectively. This result verifies the validity of the constraint controller in Lemma 2.

### 5.2. Validation Simulations of the Proposed Controller for Multi-Agent Systems

In this section, the validation simulation of the proposed method for uncertain multi-agent systems is carried out with Condition 1. The initial detector parameter is Y=4. The initial parameters are †=0.1, U=30, ${\hat{\theta }}_{l}\left(0\right)={\hat{\theta }}_{i}\left(0\right)=0$, ${\hat{k}}_{l}\left(0\right)={\hat{k}}_{i}\left(0\right)=0.4$, $\xi_{l}=\xi_{r}=\sqrt{10}\times 10^{-3}$. The actuator fault parameters in Definition 2 are set as follows: $\eta_{l,k}=\varpi_{l,k}=t_{a_{l,k}}=\infty$; $\eta_{1,k}=0.05$, $\varpi_{1,k}=1$, $t_{a_{1,k}}=20$; $\eta_{2,k}=0.05$, $\varpi_{2,k}=1.5$, $t_{a_{2,k}}=30$; $\eta_{3,k}=0.05$, $\varpi_{3,k}=0.5$, $t_{a_{3,k}}=10$.

**Figure 8:**
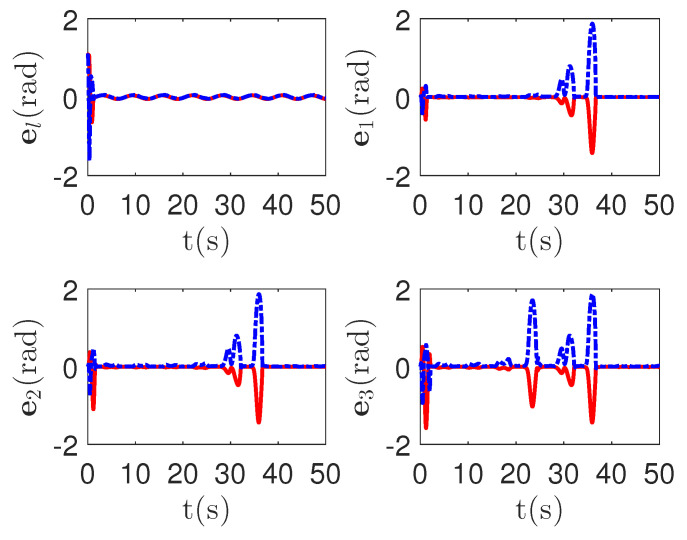
The tracking error curves of leader $e_{l}=q_{l,1}-q_{d,1}$ and followers $e_{i}=q_{i,1}-q_{l,1}$ for the proposed method with different actuator faults of subsystems. *Figure legends*: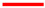: $e_{i,1},i=l,1,2,3$;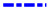: $e_{i,2},i=l,1,2,3$.

**Figure 9:**
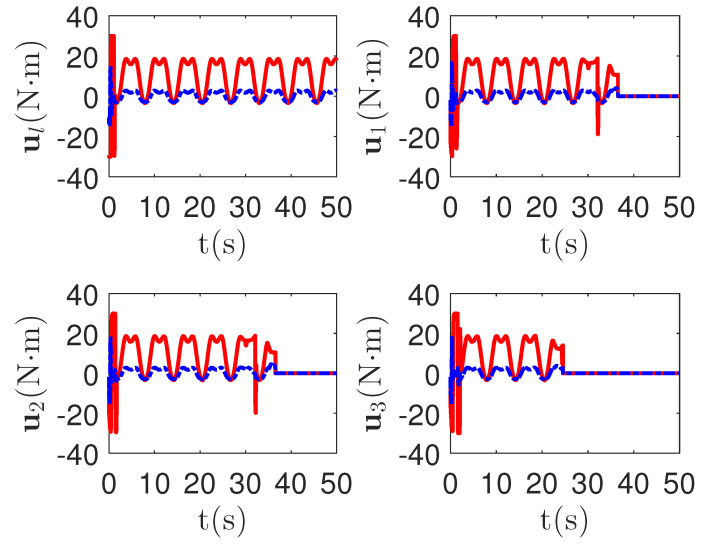
The tracking error curves of leader $u_{l}={\left[u_{l,1},u_{l,2}\right]}^{T}$ and followers $u_{i}={\left[u_{i,1},u_{i,2}\right]}^{T}$ for the proposed method with different actuator faults of subsystems. *Figure legends*: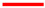: $u_{i,1},i=l,1,2,3$;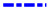: $u_{i,2},i=l,1,2,3$.

**Figure 10:**
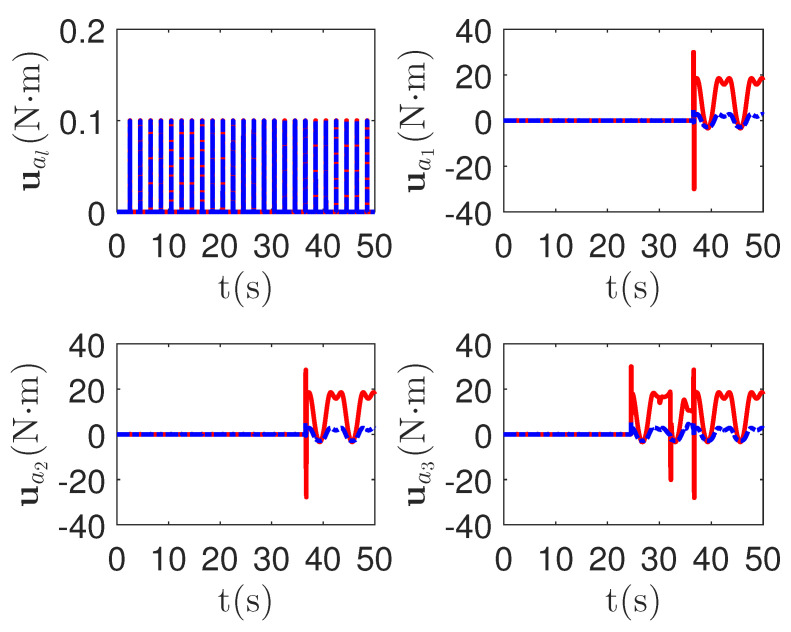
The tracking error curves of leader $u_{a_{l}}={\left[u_{a_{l,1}},u_{a_{l,2}}\right]}^{T}$ and followers $u_{a_{i}}={\left[u_{a_{i,1}},u_{a_{i,2}}\right]}^{T}$ for the proposed method with different actuator faults of subsystems. *Figure legends*: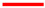: $u_{a_{i,1}},i=l,1,2,3$;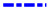: $u_{a_{i,2}},i=l,1,2,3$.

As shown in Figure 8, it is clear that the final tracking error of the proposed method is about 0.02 at 50 s, which is less than the predefined accuracy † = 0.1. According to the results in Figure 9 and Figure 10, under three different fault types, the proposed method successfully carries out the corresponding fault detection and controller reconstruction of three followers. When the sensor detects the occurrence of acceleration-level abnormal phenomena, Algorithm 1 judges the system failure flag=1 at about 36.55 s, 36.55 s and 24.55 s, respectively. Meanwhile, the detector is not triggered by mistake for the leader flag=0. Furthermore, as shown in Figure 9 and Figure 10, the maximum absolute values of $u_{i}$ and $u_{a_{i}}$ are 30.00 and 30.00, respectively. This result verifies the validity of the constraint controller in Lemma 2.

### 5.3. Comparative Simulations between the Proposed Method and the Passive Fault-Tolerant Method

In this section, comparative simulations between the proposed method and the passive fault-tolerant method [67] for uncertain multi-agent systems are carried out with Condition 1. In order to ensure the fairness of the comparison, we use the same fuzzy approximator; the only difference between them is the active detection strategy and passive fault-tolerant strategy. The initial detector parameter is Y=4. The initial parameters are †=0.1, U=30, ${\hat{\theta }}_{l}\left(0\right)={\hat{\theta }}_{i}\left(0\right)=0$, ${\hat{k}}_{l}\left(0\right)={\hat{k}}_{i}\left(0\right)=0.4$, $\xi_{l}=\xi_{r}=\sqrt{10}\times 10^{-3}$. The actuator fault parameters in Definition 2 are set as follows: $\eta_{l,k}=\eta_{i,k}=0.05$, $\varpi_{l,k}=\varpi_{i,k}=0.5$, $t_{a_{l,k}}=t_{a_{i,k}}=10$.

**Figure 11:**
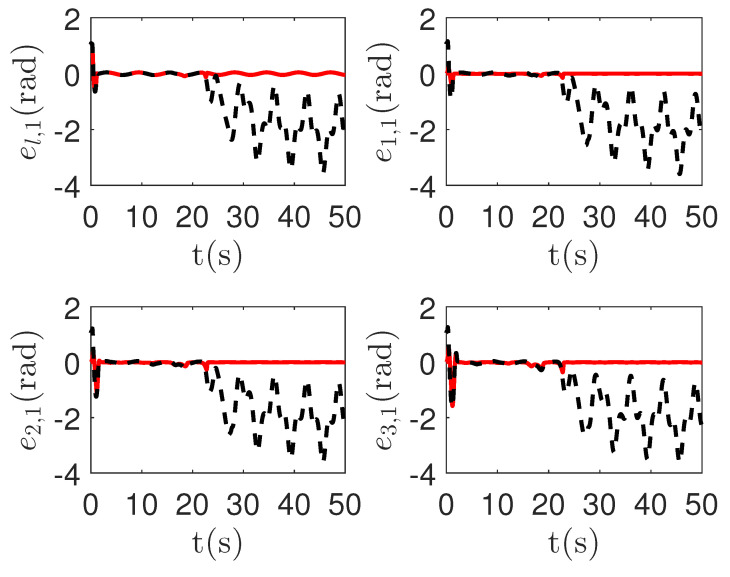
The tracking error curves of leader $e_{l,1}=q_{l,1,1}-q_{d,1,1}$ and followers $e_{i,1}=q_{i,1,1}-q_{l,1,1}$ in the proposed method and passive fault-tolerant control method [67]. *Figure legends*: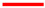: *The proposed method*;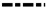: *The passive fault-tolerant control method [67]*.

**Figure 12:**
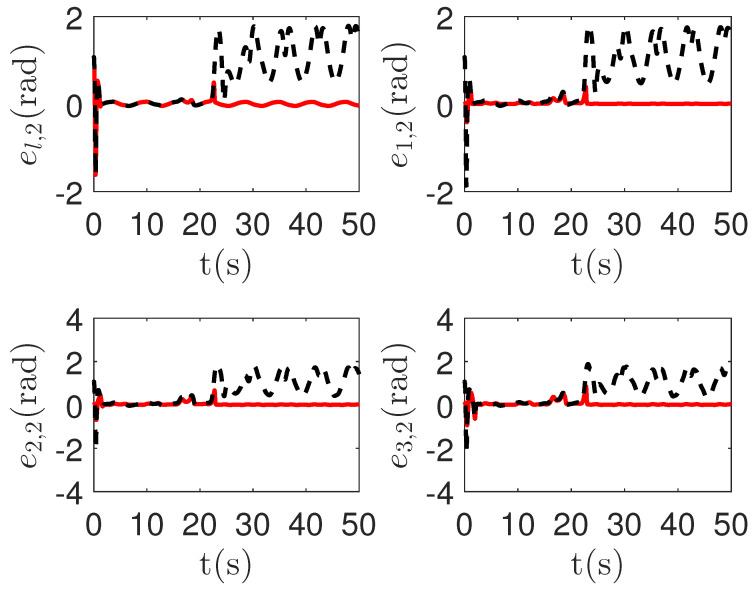
The tracking error curves of leader $e_{l,2}=q_{l,1,2}-q_{d,1,2}$ and followers $e_{i,2}=q_{i,1,2}-q_{l,1,2}$ in the proposed method and passive fault-tolerant control method [67]. *Figure legends*: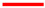: *The proposed method*;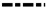: *The passive fault-tolerant control method [67]*.

As shown in Figure 11 and Figure 12, it is clear that the final tracking error of the proposed method is about 0.05 at 50 s, which is less than the predefined accuracy † = 0.1. However, the tracking error of passive fault-tolerant control method is not convergent. The reason is that passive fault-tolerant controllers can only operate under minor actuator failures. If the fault function $\Psi_{i,k}\left(q_{i,1,k},t\right)$ is too small, the more master control input is required. However, by considering the control input constraint in Condition 1, the control system can not be stable.

### 5.4. The Comparative Simulations between the Proposed Method and Active Fault-Tolerant Method

**Figure 13:**
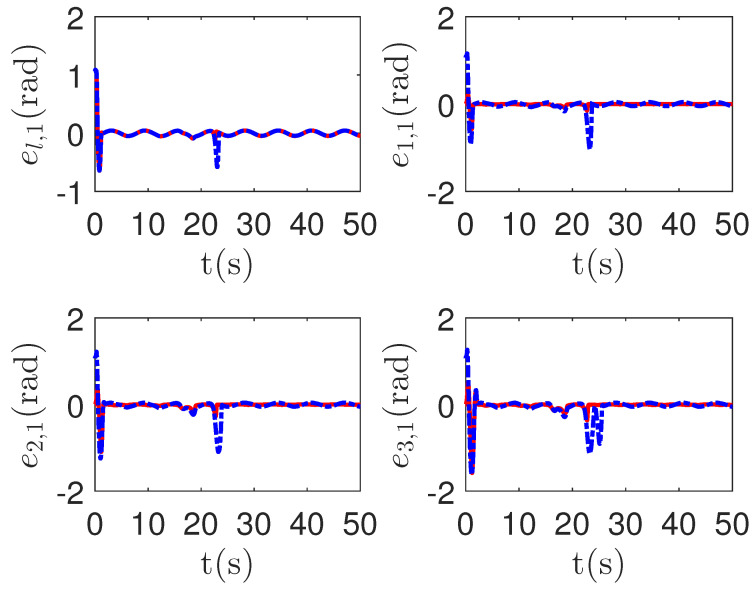
The tracking error curves of leader $e_{l,1}=q_{l,1,1}-q_{d,1,1}$ and followers $e_{i,1}=q_{i,1,1}-q_{l,1,1}$ in the proposed method and passive fault-detection control method [68]. *Figure legends*: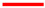: *The proposed method*;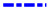: *The active fault-tolerant control method with passive fault detection [68]*.

**Figure 14:**
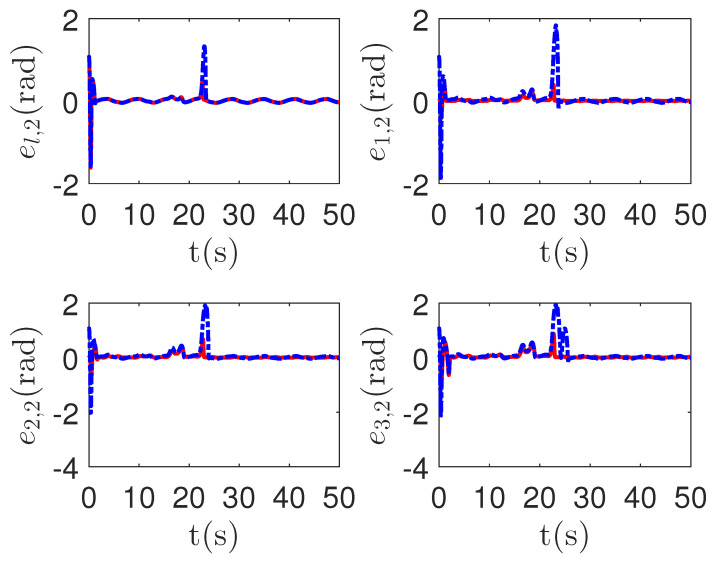
The tracking error curves of leader $e_{l,2}=q_{l,1,2}-q_{d,1,2}$ and followers $e_{i,2}=q_{i,1,2}-q_{l,1,2}$ in the proposed method and passive fault-detection control method [68]. *Figure legends*: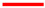: *The proposed method*;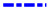: *The active fault-tolerant control method with passive fault detection [68]*.

In this section, the comparative simulations between the proposed method and active fault-tolerant method [68] for uncertain multi-agent systems is carried out with Condition 1. This active fault-tolerant method [68] adopts the passive fault-detection algorithm. In order to ensure the fairness of the comparison, we use the same main controller and adjust similar tracking accuracy. The only difference is that the active and passive detection mechanism. As shown in Figure 13 and Figure 14, the final tracking errors of the two algorithms are similar. The initial detector parameter is Y=4. The initial parameters are † = 0.1, U = 30, ${\hat{\theta }}_{l}\left(0\right)={\hat{\theta }}_{i}\left(0\right)=0$, ${\hat{k}}_{l}\left(0\right)={\hat{k}}_{i}\left(0\right)=0.4$, $\xi_{l}=\xi_{r}=\sqrt{10}\times 10^{-3}$. The actuator fault parameters in Definition 2 are set as follows: $\eta_{l,k}=\eta_{i,k}=0.05$, $\varpi_{l,k}=\varpi_{i,k}=0.5$, $t_{a_{l,k}}=t_{a_{i,k}}=10$.

**Figure 15:**
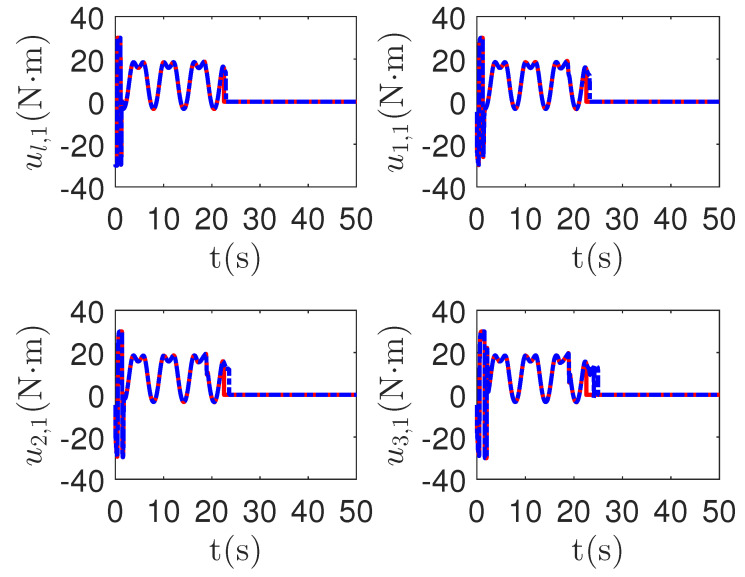
The main control input curves of leader $u_{l,1}$ and followers $u_{i,1}$ in the proposed method and passive fault-detection control method [68]. *Figure legends*: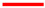: *The proposed method*;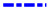: *The active fault-tolerant control method with passive fault detection [68]*.

**Figure 16:**
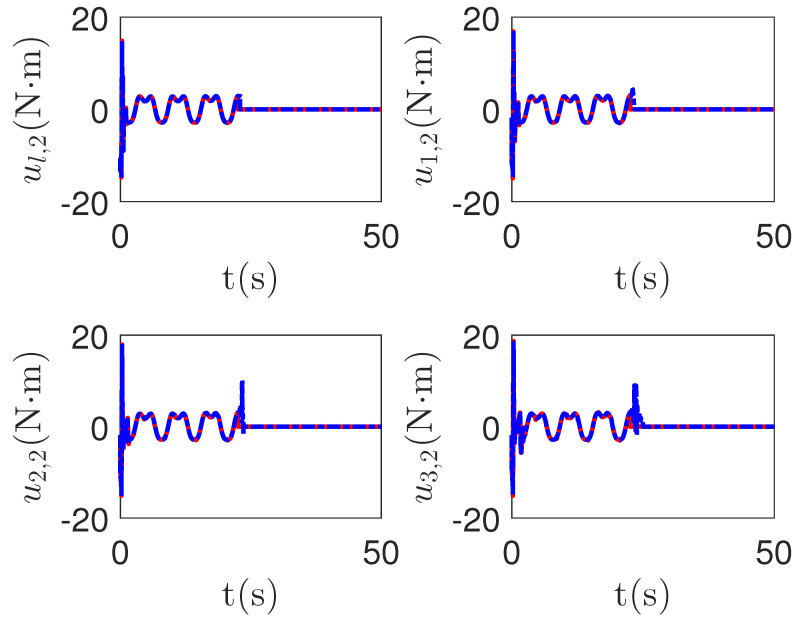
The main control input curves of leader $u_{l,2}$ and followers $u_{i,2}$ in the proposed method and passive fault-detection control method [68]. *Figure legends*: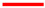: *The proposed method*;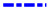: *The active fault-tolerant control method with passive fault detection [68]*.

**Figure 17:**
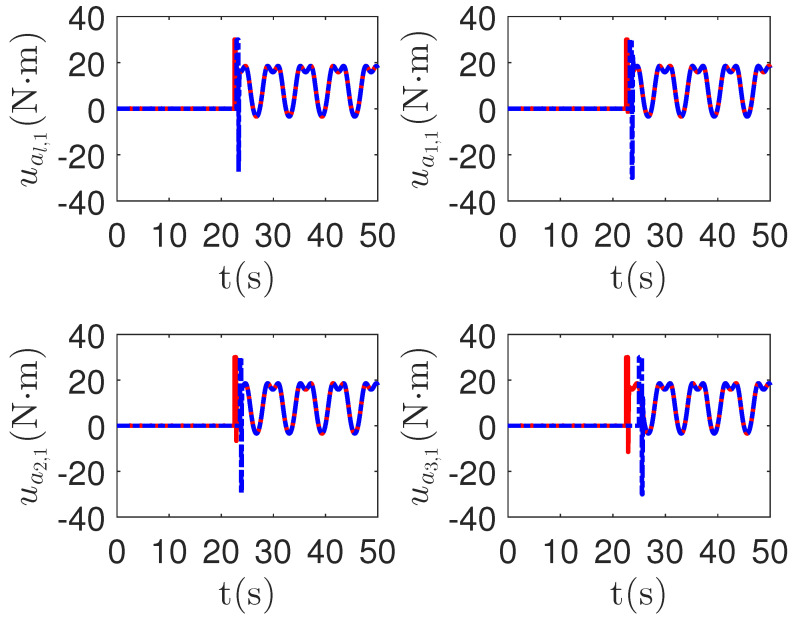
The redundant control input curves of leader $u_{a_{l,1}}$ and followers $u_{a_{i,1}}$ in the proposed method and passive fault-detection control method [68]. *Figure legends*: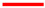: *The proposed method*;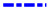: *The active fault-tolerant control method with passive fault detection [68]*.

**Figure 18:**
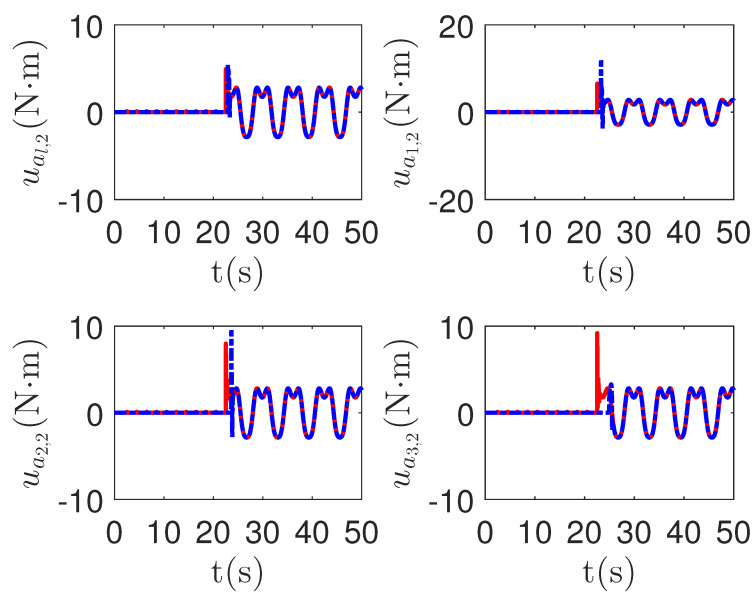
The redundant control input curves of leader $u_{a_{l,2}}$ and followers $u_{a_{i,2}}$ in the proposed method and passive fault-detection control method [68]. *Figure legends*: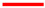: *The proposed method*;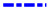: *The active fault-tolerant control method with passive fault detection [68]*.

As shown in Figure 13 and Figure 14, when the switch happens, the maximum absolute values of tracking errors in the proposed control method are [0.12, 0.49]*^T^*, [0.15, 0.39]*^T^*, [0.27, 0.67]*^T^* and [0.36, 0.86]*^T^*, respectively. When the switch happens, the maximum absolute values of tracking errors in the compared control method [68] are [0.58, 1.33]*^T^*, [1.04, 1.84]*^T^*, [1.09, 1.91]*^T^* and [1.13, 1.94]*^T^*, respectively. It is clear that the system tracking performance of the proposed method is better than that of the passive detection method when switching occurs. According to the results in Figure 15 and Figure 16, the chattering of the proposed main controller is weaker than that of the compared control method during switching. In Figure 17 and Figure 18, the chattering of the proposed main controller is basically weaker than that of the compared control method during switching. Furthermore, according to Figure 15, Figure 16, Figure 17 and Figure 18, under the same fault, the proposed active fault detector basically detects the fault occurrence at 22.55 s. The compared passive fault detector detects the fault occurrence at 22.91 s, 23.25 s, 23.60 s and 24.90, respectively. This simulation result means that passive detection is more susceptible to topology communication interference of multi-agent systems than active detection.

## 6. Conclusions

A novel adaptive interval Type-II fuzzy fault-tolerant control method was proposed for constrained uncertain 2-DOF robotic multi-agent systems by considering an active fault-detection algorithm. This control method can realize the predefined-accuracy stability of multi-agent systems under input saturation, complex actuator failure and high-order system uncertainties. Firstly, a novel active fault-detection algorithm based on pulse-wave function was proposed to detect the failure time of multi-agent systems for the first time. Compared with the existing passive fault-detection methods, the novel active detection algorithm can resist more topology communication interference than passive detection. Then, an improved fault-tolerant control algorithm was adopted to deal with more complex actuator failures. In the end, based on the interval Type-II fuzzy approximated system, a novel adaptive fuzzy fault-tolerant controller was proposed for constrained uncertain mechanical multi-agent systems to achieve predefined-accuracy stability. Compared with other fault-tolerant control methods, the proposed method can achieve predefined-accuracy stability of multi-agent systems under complex multi-agent faults. Meanwhile, the switching chattering of the controller was weaker. These theoretical results were verified by simulation.

## Data Availability

Data sharing not applicable.

## Appendix A

**Graph theory** [66]: The topological structure of a multi-agent system is represented by one directed graph G=(V,E), where $V=\left\{\begin{array}{ccc} v_{1} & \ldots & v_{n} \end{array}\right\}$ and E⊆V×V. $v_{i}$ is a node which represents agent *i*. If $v_{i}$ only gets information from $v_{j}$, $e_{ji}=\left(v_{j},v_{i}\right)\in E$ can be true. $v_{j}$ represents the neighbor of $v_{i}$. $N_{i}=\left\{v_{j}\left|(v_{j},v_{i})\in E\right.\right\}$ represents a collection of $v_{j}$. If weights are considered, the proposed graph is a weighted graph. Adjacency matrix $W_{1}=\left[w_{1,ij}\right]\in R^{N\times N}$ is also the weighted topology. $w_{1,ij}>0$ if $e_{ji}=\left(v_{j},v_{i}\right)\in E$, otherwise $w_{1,ij}=0$. $D=diag\left(d_{1},d_{2},\ldots ,d_{N}\right)\in R^{N\times N}$, where $d_{i}=\sum_{j=1}^{N}w_{1,ij}$ is in-degree of $v_{i}$. The Laplacian matrix is L=D−A. The communication topology is described by augmented graph $\bar{G}=\left(\bar{V},\bar{E}\right)$. There are one leader and *N* followers.

## Appendix B

**Proof.** Step 1: Consider a candidate Lyapunov function:

(A1) $$\begin{array}{c} V_{l,1}=\frac{z_{l,1}^{T}z_{l,1}}{2} \end{array}$$

By considering (13) and (14), the time derivative of Lyapunov function (A1) can be deduced as:

(A2) $$\begin{array}{c} {\dot{V}}_{l,1}=z_{l,1}^{T}{\dot{z}}_{l,1}\\ \;\;\;\;\;\;=z_{l,1}^{T}\left(q_{l,2}-{\dot{q}}_{d_{l,1}}\right)\\ \;\;\;\;\;\;=z_{l,1}^{T}\left(z_{l,2}+\alpha_{l,1}-{\dot{q}}_{d_{l,1}}\right)\\ \;\;\;\;\;\;=-\frac{\left(2{†}^{2}+{\hat{k}}_{l}\right)z_{l,1}^{T}z_{l,1}}{2{†}^{2}}+z_{l,1}^{T}z_{l,2} \end{array}$$

Step 2: Consider a candidate Lyapunov function:

(A3) $$\begin{array}{c} V_{l,2}=V_{l,1}+\frac{z_{l,2}^{T}z_{l,2}}{2}+\frac{{\tilde{\theta }}_{l}^{T}{\tilde{\theta }}_{l}}{2}+\frac{\xi_{l}^{2}{\tilde{k}}_{l}^{2}}{2} \end{array}$$

where ${\tilde{\theta }}_{l}=\theta_{l}-{\hat{\theta }}_{l}$. ${\tilde{k}}_{l}=k_{l}-{\hat{k}}_{l}$, in which $k_{l}\geq \delta_{l}^{T}\delta_{l}+1$ is defined in (A7). By considering Lemma 2, (7) and (13), the time derivative of Lyapunov function (A3) can be deduced as:

(A4) $$\begin{array}{c} {\dot{V}}_{l,2}=-\frac{\left(2{†}^{2}+{\hat{k}}_{l}\right)z_{l,1}^{T}z_{l,1}}{2{†}^{2}}+z_{l,1}^{T}z_{l,2}+z_{l,2}^{T}{\dot{z}}_{l,2}+{\tilde{\theta }}_{l}^{T}\left(-{\dot{\hat{\theta }}}_{l}\right)+\xi_{l}^{2}{\tilde{k}}_{l}\left(-{\dot{\hat{k}}}_{l}\right)\\ \;\;\;\;\;\;=-\frac{\left(2{†}^{2}+{\hat{k}}_{l}\right)z_{l,1}^{T}z_{l,1}}{2{†}^{2}}+z_{l,1}^{T}z_{l,2}+z_{l,2}^{T}\left[f_{l}\left(q_{l,1},q_{l,2}\right)+\Delta f_{l}\left(q_{l,1},q_{l,2}\right)\right.\\ \;\;\;\;\;\;\;\;\;\;\;+\left.\left(g_{l}\left(q_{l,1}\right)+\Delta g_{l}\left(q_{l,1}\right)\right)\left(v_{l}-e\left(v_{l}\right)+u_{a_{l}}\right)-{\dot{\alpha }}_{l,1}\right]-{\tilde{\theta }}_{l}^{T}{\dot{\hat{\theta }}}_{l}-\xi_{l}^{2}{\tilde{k}}_{l}{\dot{\hat{k}}}_{l} \end{array}$$

where $e\left(v_{l}\right)={\left[e_{1}\left(v_{l,1}\right),\ldots ,e_{n}\left(v_{l,n}\right)\right]}^{T}$. By considering (14), (A4) can be deduced as:

(A5) $$\begin{array}{c} {\dot{V}}_{l,2}=-\frac{\left(2{†}^{2}+{\hat{k}}_{l}\right)z_{l,1}^{T}z_{l,1}}{2{†}^{2}}+z_{l,1}^{T}z_{l,2}+z_{l,2}^{T}\left[f_{l}\left(q_{l,1},q_{l,2}\right)+\Delta f_{l}\left(q_{l,1},q_{l,2}\right)+\Delta g_{l}\left(q_{l,1}\right)(v_{l}\right.\\ \;\;\;\;\;\;\;\;\;\;\;-e\left(v_{l}\right)+u_{a_{l}})+g_{l}\left(q_{l,1}\right)\left[-g_{l}{\left(q_{l,1}\right)}^{-1}\left(f_{l}\left(q_{l,1},q_{l,2}\right)+\frac{1}{2}{\hat{\theta }}_{l}^{T}\psi_{l}\left(q_{l,1},q_{l,2}\right)z_{l,2}\right.\right.\\ \;\;\;\;\;\;\;\;\;\;\;+\left.\left.\left.z_{l,1}+\frac{3}{2}z_{l,2}-{\dot{\alpha }}_{l,1}\right)-e\left(v_{l}\right)+u_{a_{l}}\right]-{\dot{\alpha }}_{l,1}\right]-{\tilde{\theta }}_{l}^{T}{\dot{\hat{\theta }}}_{l}-\xi_{l}^{2}{\tilde{k}}_{l}{\dot{\hat{k}}}_{l}\\ \;\;\;\;\;\;=-\frac{\left(2{†}^{2}+{\hat{k}}_{l}\right)z_{l,1}^{T}z_{l,1}}{2{†}^{2}}+z_{l,2}^{T}\left[\Delta f_{l}\left(q_{l,1},q_{l,2}\right)+\Delta g_{l}\left(q_{l,1}\right)\left(v_{l}-e\left(v_{l}\right)+u_{a_{l}}\right)\right.\\ \;\;\;\;\;\;\;\;\;\;\;+\left.g_{l}\left(q_{l,1}\right)\left(u_{a_{l}}-e\left(v_{l}\right)\right)\right]-\frac{1}{2}z_{l,2}^{T}{\hat{\theta }}_{l}^{T}\psi_{l}\left(q_{l,1},q_{l,2}\right)z_{l,2}-\frac{3}{2}z_{l,2}^{T}z_{l,2}-{\tilde{\theta }}_{l}^{T}{\dot{\hat{\theta }}}_{l}-\xi_{l}^{2}{\tilde{k}}_{l}{\dot{\hat{k}}}_{l}\\ \;\;\;\;\;\;\leq -\frac{\left(2{†}^{2}+{\hat{k}}_{l}\right)z_{l,1}^{T}z_{l,1}}{2{†}^{2}}-\frac{3}{2}z_{l,2}^{T}z_{l,2}+{\left|z_{l,2}\right|}^{T}\chi \left(q_{l,1},q_{l,2}\right)\\ \;\;\;\;\;\;\;\;\;\;\;-\frac{1}{2}z_{l,2}^{T}z_{l,2}{\hat{\theta }}_{l}^{T}\psi_{l}\left(q_{l,1},q_{l,2}\right)-{\tilde{\theta }}_{l}^{T}{\dot{\hat{\theta }}}_{l}-\xi_{l}^{2}{\tilde{k}}_{l}{\dot{\hat{k}}}_{l} \end{array}$$

in which

(A6) $$\begin{array}{c} \chi \left(q_{l,1},q_{l,2}\right)=\left|\Delta f_{l}\left(q_{l,1},q_{l,2}\right)\right|+\left|\Delta g_{l}\left(q_{l,1}\right)\right|\left(U+E+U\right)+\left|g_{l}\left(q_{l,1}\right)\right|\left(U_{m}+E\right) \end{array}$$

where $\left|\cdot \right|$ stands for taking the absolute value of each element in the vector or matrix. $U={\left[U,\ldots ,U\right]}_{1\times n}^{T}$ and $E={\left[E,\ldots ,E\right]}_{1\times n}^{T}$ are constant vectors. $U_{m}={\left[U_{m},\ldots ,U_{m}\right]}^{T}$. According to Lemma 1, $\chi (q_{l,1},q_{l,2})$ can be approximated by an interval Type-II fuzzy logic system:

(A7) $$\begin{array}{c} \chi_{k}\left(q_{l,1},q_{l,2}\right)=w_{l,k}^{T}\beta_{l,k}\left(q_{l,1},q_{l,2}\right)+\delta_{k}\left(q_{l,1},q_{l,2}\right) \end{array}$$

where $\chi \left(q_{l,1},q_{l,2}\right)={\left[\chi_{1}\left(q_{l,1},q_{l,2}\right),\ldots ,\chi_{n}\left(q_{l,1},q_{l,2}\right)\right]}^{T}$. $\left|\delta_{k}\left(q_{l,1},q_{l,2}\right)\right|\leq \delta_{l,k}$. $\delta_{l,k}$ is an arbitrary small bounded parameter and the boundary is defined as: $\delta_{l}^{T}\delta_{l}+1\leq k_{l}$. $\delta_{l}={\left[\delta_{l,1},\ldots ,\delta_{l,n}\right]}^{T}$. Then, by considering AM–GM inequality, matrix transformation and (A7), (A5) can be written as:

(A8) $$\begin{array}{c} {\dot{V}}_{l,2}\leq -\frac{\left(2{†}^{2}+{\hat{k}}_{l}\right)z_{l,1}^{T}z_{l,1}}{2{†}^{2}}-\frac{3}{2}z_{l,2}^{T}z_{l,2}+\frac{1^{2}}{2}+\frac{z_{l,2}^{T}z_{l,2}\theta_{l}^{T}\psi_{l}\left(q_{l,1},q_{l,2}\right)}{2}+\frac{z_{l,2}^{T}z_{l,2}}{2}+\frac{\delta_{l}^{T}\delta_{l}}{2}\\ \;\;\;\;\;\;\;\;\;\;\;-\frac{1}{2}z_{l,2}^{T}z_{l,2}\left(\theta_{l}^{T}-{\tilde{\theta }}_{l}^{T}\right)\psi_{l}\left(q_{l,1},q_{l,2}\right)-{\tilde{\theta }}_{l}^{T}{\dot{\hat{\theta }}}_{l}-\xi_{l}^{2}{\tilde{k}}_{l}{\dot{\hat{k}}}_{l} \end{array}$$

where $\theta_{l,k}=w_{l,k}^{T}w_{l,k}$ and $\theta_{l}={\left[\theta_{l,1},\ldots ,\theta_{l,n}\right]}^{T}$. Then, by substituting (15), (A8) can be written as:

(A9) $$\begin{array}{c} {\dot{V}}_{l,2}\leq -\frac{\left(2{†}^{2}+{\hat{k}}_{l}\right)z_{l,1}^{T}z_{l,1}}{2{†}^{2}}-z_{l,2}^{T}z_{l,2}+\frac{1}{2}+\frac{\delta_{l}^{T}\delta_{l}}{2}\\ \;\;\;\;\;\;\;\;\;\;\;+\frac{z_{l,2}^{T}z_{l,2}{\tilde{\theta }}_{l}^{T}\psi_{l}\left(q_{l,1},q_{l,2}\right)}{2}-{\tilde{\theta }}_{l}^{T}\left(\frac{z_{l,2}^{T}z_{l,2}\psi_{l}\left(q_{l,1},q_{l,2}\right)}{2}\right)-\xi_{l}^{2}{\tilde{k}}_{l}{\dot{\hat{k}}}_{l}\\ \;\;\;\;\;\;\leq -z_{l,1}^{T}z_{l,1}-z_{l,2}^{T}z_{l,2}-\frac{\left(k_{l}-{\tilde{k}}_{l}\right)z_{l,1}^{T}z_{l,1}}{2{†}^{2}}+\frac{1+\delta_{l}^{T}\delta_{l}}{2}-\xi_{l}^{2}{\tilde{k}}_{l}{\dot{\hat{k}}}_{l} \end{array}$$

Then, by substituting (16), (A9) can be written as:

(A10) $$\begin{array}{c} {\dot{V}}_{l,2}\leq -z_{l,1}^{T}z_{l,1}-z_{l,2}^{T}z_{l,2}+\frac{k_{l}}{2}\left(1-\frac{z_{l,1}^{T}z_{l,1}}{{†}^{2}}\right)+\frac{{\tilde{k}}_{l}z_{l,1}^{T}z_{l,1}}{2{†}^{2}}-\xi_{l}^{2}{\tilde{k}}_{l}\frac{z_{l,1}^{T}z_{l,1}}{2\xi_{l}^{2}{†}^{2}}\\ \;\;\;\;\;\;\leq -z_{l,1}^{T}z_{l,1}-z_{l,2}^{T}z_{l,2}+\frac{k_{l}}{2}\left(1-\frac{z_{l,1}^{T}z_{l,1}}{{†}^{2}}\right) \end{array}$$

According to (A10), it is clear that $z_{l,1}$ can converge to a neighborhood of zero $B_{l}=\left\{\left.z_{l,1}\right|∥z_{l,1}∥\leq †\right\}$ if there is no actuator fault. The proof is completed. □

## Appendix C

**Proof.** Step 1: Consider a candidate Lyapunov function:

(A11) $$\begin{array}{c} V_{i,1}=\frac{z_{i,1}^{T}z_{i,1}}{2} \end{array}$$

By considering (8), the time derivative of Lyapunov function (A11) can be deduced as:

(A12) $$\begin{array}{c} {\dot{V}}_{i,1}=z_{i,1}^{T}{\dot{z}}_{i,1}\\ \;\;\;\;\;\;=z_{i,1}^{T}\left[\left(b_{i}+\sum_{h=1}^{N}a_{ih}\right)\left(z_{i,2}+\alpha_{i,1}\right)-b_{i}q_{l,2}-\sum_{h=1}^{N}a_{ih}q_{h,2}\right] \end{array}$$

By considering (18), (A12) can be deduced as:

(A13) $$\begin{array}{c} {\dot{V}}_{i,1}=z_{i,1}^{T}\left[\left(b_{i}+\sum_{h=1}^{N}a_{ih}\right)\left(z_{i,2}+\frac{1}{\left(b_{i}+\sum_{h=1}^{N}a_{ih}\right)}\left(b_{i}q_{l,2}+\sum_{h=1}^{N}a_{ih}q_{h,2}-\frac{\left(2{†}^{2}+{\hat{k}}_{i}\right)z_{i,1}}{2{†}^{2}}\right)\right)\right.\\ \;\;\;\;\;\;\;\;\;\;\;-\left.b_{i}q_{l,2}-\sum_{h=1}^{N}a_{ih}q_{h,2}\right]\\ \;\;\;\;\;\;=-\frac{\left(2{†}^{2}+{\hat{k}}_{i}\right)z_{i,1}^{T}z_{i,1}}{2{†}^{2}}+\left(b_{i}+\sum_{h=1}^{N}a_{ih}\right)z_{i,1}^{T}z_{i,2} \end{array}$$

Step 2: Consider a candidate Lyapunov function:

(A14) $$\begin{array}{c} V_{i,2}=V_{i,1}+\frac{z_{i,2}^{T}z_{i,2}}{2}+\frac{{\tilde{\theta }}_{i}^{T}{\tilde{\theta }}_{i}}{2}+\frac{\xi_{i}^{2}{\tilde{k}}_{i}^{2}}{2} \end{array}$$

where ${\tilde{\theta }}_{i}=\theta_{i}-{\hat{\theta }}_{i}$. ${\tilde{k}}_{i}=k_{i}-{\hat{k}}_{i}$, in which $k_{i}\geq \delta_{i}^{T}\delta_{i}+1$ is defined in (A18). By considering Lemma 2, (6) and (17), the time derivative of Lyapunov function (A14) can be deduced as:

(A15) $$\begin{array}{c} {\dot{V}}_{i,2}=-\frac{\left(2{†}^{2}+{\hat{k}}_{i}\right)z_{i,1}^{T}z_{i,1}}{2{†}^{2}}+\left(b_{i}+\sum_{h=1}^{N}a_{ih}\right)z_{i,1}^{T}z_{i,2}+z_{i,2}^{T}{\dot{z}}_{i,2}+{\tilde{\theta }}_{i}^{T}\left(-{\dot{\hat{\theta }}}_{i}\right)+\xi_{i}^{2}{\tilde{k}}_{i}\left(-{\dot{\hat{k}}}_{i}\right)\\ \;\;\;\;\;\;=-\frac{\left(2{†}^{2}+{\hat{k}}_{i}\right)z_{i,1}^{T}z_{i,1}}{2{†}^{2}}+\left(b_{i}+\sum_{h=1}^{N}a_{ih}\right)z_{i,1}^{T}z_{i,2}+z_{i,2}^{T}\left[f_{i}\left(q_{i,1},q_{i,2}\right)+\Delta f_{i}\left(q_{i,1},q_{i,2}\right)\right.\\ \;\;\;\;\;\;\;\;\;\;\;+\left.\left(g_{i}\left(q_{i,1}\right)+\Delta g_{i}\left(q_{i,1}\right)\right)\left(v_{i}-e\left(v_{i}\right)+u_{a_{i}}\right)-{\dot{\alpha }}_{i,1}\right]-{\tilde{\theta }}_{i}^{T}{\dot{\hat{\theta }}}_{i}-\xi_{i}^{2}{\tilde{k}}_{i}{\dot{\hat{k}}}_{i} \end{array}$$

where $e\left(v_{i}\right)={\left[e_{1}\left(v_{i,1}\right),\ldots ,e_{n}\left(v_{i,n}\right)\right]}^{T}$. By considering (18), (A15) can be deduced as:

(A16) $$\begin{array}{c} {\dot{V}}_{i,2}=-\frac{\left(2{†}^{2}+{\hat{k}}_{i}\right)z_{i,1}^{T}z_{i,1}}{2{†}^{2}}+\left(b_{i}+\sum_{h=1}^{N}a_{ih}\right)z_{i,1}^{T}z_{i,2}+z_{i,2}^{T}\left[f_{i}\left(q_{i,1},q_{i,2}\right)\right.\\ \;\;\;\;\;\;\;\;\;\;\;+\Delta f_{i}\left(q_{i,1},q_{i,2}\right)+\Delta g_{i}\left(q_{i,1}\right)\left(v_{i}-e\left(v_{i}\right)+u_{a_{i}}\right)\\ \;\;\;\;\;\;\;\;\;\;\;+g_{i}\left(q_{i,1}\right)\left[-g_{i}{\left(q_{i,1}\right)}^{-1}\left(f_{i}\left(q_{i,1},q_{i,2}\right)+\frac{1}{2}{\hat{\theta }}_{i}^{T}\psi_{i}\left(q_{i,1},q_{i,2}\right)z_{i,2}\right.\right.\\ \;\;\;\;\;\;\;\;\;\;\;+\left.\left.\left.\left(b_{i}+\sum_{h=1}^{N}a_{ih}\right)z_{i,1}+\frac{3}{2}z_{i,2}-{\dot{\alpha }}_{i,1}\right)-e\left(v_{i}\right)+u_{a_{i}}\right]-{\dot{\alpha }}_{i,1}\right]-{\tilde{\theta }}_{i}^{T}{\dot{\hat{\theta }}}_{i}-\xi_{i}^{2}{\tilde{k}}_{i}{\dot{\hat{k}}}_{i}\\ \;\;\;\;\;\;\leq -\frac{\left(2{†}^{2}+{\hat{k}}_{i}\right)z_{i,1}^{T}z_{i,1}}{2{†}^{2}}-\frac{3}{2}z_{i,2}^{T}z_{i,2}+{\left|z_{i,2}\right|}^{T}\chi \left(q_{i,1},q_{i,2}\right)\\ \;\;\;\;\;\;\;\;\;\;\;-\frac{1}{2}z_{i,2}^{T}z_{i,2}{\hat{\theta }}_{i}^{T}\psi_{i}\left(q_{i,1},q_{i,2}\right)-{\tilde{\theta }}_{i}^{T}{\dot{\hat{\theta }}}_{i}-\xi_{i}^{2}{\tilde{k}}_{i}{\dot{\hat{k}}}_{i} \end{array}$$

in which

(A17) $$\begin{array}{c} \chi \left(q_{i,1},q_{i,2}\right)=\left|\Delta f_{i}\left(q_{i,1},q_{i,2}\right)\right|+\left|\Delta g_{i}\left(q_{i,1}\right)\right|\left(U+E+U\right)+\left|g_{i}\left(q_{i,1}\right)\right|\left(u_{m}+e\right) \end{array}$$

where $\left|\cdot \right|$ stands for taking the absolute value of each element in the vector or matrix. $U={\left[U,\ldots ,U\right]}_{1\times n}^{T}$ and $E={\left[E,\ldots ,E\right]}_{1\times n}^{T}$ are the constant vectors. $U_{m}={\left[U_{m},\ldots ,U_{m}\right]}^{T}$. According to Lemma 1, $\chi (q_{i,1},q_{i,2})$ can be approximated by an interval Type-2 fuzzy logic system:

(A18) $$\begin{array}{c} \chi_{k}\left(q_{i,1},q_{i,2}\right)=w_{i,k}^{T}\beta_{i,k}\left(q_{i,1},q_{i,2}\right)+\delta_{k}\left(q_{i,1},q_{i,2}\right) \end{array}$$

where $\chi \left(q_{i,1},q_{i,2}\right)={\left[\chi_{1}\left(q_{i,1},q_{i,2}\right),\ldots ,\chi_{n}\left(q_{i,1},q_{i,2}\right)\right]}^{T}$. $\left|\delta_{k}\left(q_{i,1},q_{i,2}\right)\right|\leq \delta_{i,k}$. $\delta_{i,k}$ is an arbitrary small bounded parameter and the boundary is defined as: $\delta_{i}^{T}\delta_{i}+1\leq k_{i}$. $\delta_{i}={\left[\delta_{i,1},\ldots ,\delta_{i,n}\right]}^{T}$. Then, by considering AM–GM inequality, matrix transformation and (A18), (A16) can be written as:

(A19) $$\begin{array}{c} {\dot{V}}_{i,2}\leq -\frac{\left(2{†}^{2}+{\hat{k}}_{i}\right)z_{i,1}^{T}z_{i,1}}{2{†}^{2}}-\frac{3}{2}z_{i,2}^{T}z_{i,2}+\frac{1^{2}}{2}+\frac{z_{i,2}^{T}z_{i,2}\theta_{i}^{T}\psi_{i}\left(q_{i,1},q_{i,2}\right)}{2}+\frac{z_{i,2}^{T}z_{i,2}}{2}+\frac{\delta_{i}^{T}\delta_{i}}{2}\\ \;\;\;\;\;\;\;\;\;\;\;-\frac{1}{2}z_{i,2}^{T}z_{i,2}\left(\theta_{i}^{T}-{\tilde{\theta }}_{i}^{T}\right)\psi_{i}\left(q_{i,1},q_{i,2}\right)-{\tilde{\theta }}_{i}^{T}{\dot{\hat{\theta }}}_{i}-\xi_{i}^{2}{\tilde{k}}_{i}{\dot{\hat{k}}}_{i} \end{array}$$

where $\theta_{i,k}=w_{i,k}^{T}w_{i,k}$ and $\theta_{i}={\left[\theta_{i,1},\ldots ,\theta_{i,n}\right]}^{T}$. Then, by substituting (19), (A19) can be written as:

(A20) $$\begin{array}{c} {\dot{V}}_{i,2}\leq -z_{i,1}^{T}z_{i,1}-z_{i,2}^{T}z_{i,2}-\frac{\left(k_{i}-{\tilde{k}}_{i}\right)z_{i,1}^{T}z_{i,1}}{2{†}^{2}}+\frac{1+\delta_{i}^{T}\delta_{i}}{2}-\xi_{i}^{2}{\tilde{k}}_{i}{\dot{\hat{k}}}_{i} \end{array}$$

Then, by substituting (20), (A20) can be written as:

(A21) $$\begin{array}{c} {\dot{V}}_{i,2}\leq -z_{i,1}^{T}z_{i,1}-z_{i,2}^{T}z_{i,2}+\frac{k_{i}}{2}\left(1-\frac{z_{i,1}^{T}z_{i,1}}{{†}^{2}}\right) \end{array}$$

According to (A21), it is clear that $z_{i,1}$ can converge to a neighborhood of zero $B_{i}=\left\{\left.z_{i,1}\right|∥z_{i,1}∥\leq †\right\}$ if there is no actuator fault. The proof is completed. □
